# The Fundamental Neurobiological Mechanism of Oxidative Stress-Related 4E-BP2 Protein Deamidation

**DOI:** 10.3390/ijms252212268

**Published:** 2024-11-15

**Authors:** Davis Joseph

**Affiliations:** 1 Faculty of Medicine, McGill University, Montreal, QC H3A 0G4, Canada; davisandrejoseph@gmail.com or djoseph@flogen.com; 2 Flogen Technologies Inc., Mount Royal, QC H3P 2T1, Canada

**Keywords:** 4E-BP2, deamidation, Alzheimer’s, Parkinson’s, oxidative stress, neurodegeneration

## Abstract

Memory impairment is caused by the absence of the 4E-BP2 protein in the brain. This protein undergoes deamidation spontaneously in the neurons. 4E-BP2 deamidation significantly alters protein synthesis in the neurons and affects the balance of protein production required for a healthy nervous system. Any imbalance in protein production in the nervous system causes neurodegenerative diseases. Discovering what causes 4E-BP2 deamidation will make it possible to control this balance of protein production and develop effective treatments against neurodegenerative diseases such as Alzheimer’s and Parkinson’s. The purpose of this work is to discover the neurobiological mechanism that causes the deamidation reaction in the 4E-BP2 protein by performing immunoblotting in the retinal ganglia, the optic nerve, the dorsal root ganglia, the sciatic nerve, and the whole brain, extracted via dissection from 2-month-old, Wild-type male mice. The results show that axons and their unique properties cause neuron-specific 4E-BP2 deamidation in the nervous system, confirming conclusively that axons are the critical factors behind the fundamental neurobiological mechanism of 4E-BP2 protein deamidation.

## 1. Introduction

In 1976, the eIF4E protein (eukaryotic translation initiation factor 4E) was discovered [1]. This protein binds to the 7-methylguanosine 5′ cap structure (m7GpppN) of the mRNA, forms the eIF4F complex and, as a result, directs ribosomes to the 5′ cap structure of mRNA. This process initiates the translation of proteins [1]. In 1994, the 4E-binding proteins (4E-BP) were discovered [2]. These proteins bind to the eIF4E protein, inhibit the binding of eIF4G to eIF4E, and therefore prevent the formation of the eIF4F complex and the overall translation of proteins [2]. In 1999 and 2005, deamidation, which consists of converting an asparagine (Asn) to an aspartic acid, was associated with oxidative stress [3,4]. Deamidation was later characterized as a recycling mechanism for proteins damaged by oxidative stress [5]. In 2007, it was discovered that 4E-BP2, which is the dominant paralog of 4E-BPs in brain tissue, undergoes deamidation in neurons [6]. The deamidation of 4E-BP2 decreases its affinity for eIF4E and increases it for the raptor component of mTORC1. Deamidation occurs 18 days after birth in the mouse brain. Before 18 days, the protein is phosphorylated instead of deamidated in this region. Deamidation was also found to increase with increasing pH [6]. Deamidation occurs in asparagines 99 and 102 in 4E-BP2 18 days after birth [6]. Deamidation occurrence is observed in western blots by the appearance of three bands on the blot using 4E-BP2 antibodies. The bottom band represents un-deamidated 4E-BP2, the middle band represents 4E-BP2, where either Asn 99 or Asn 102 is deamidated, and the top band represents 4E-BP2, where both asparagines are deamidated [6]. In 2017, it was claimed that 4E-BP2 deamidation was also found in muscle cells [7].

However, since 2007, when it was discovered that 4E-BP2 undergoes deamidation, there has been no credible explanation for why deamidation of 4E-BP2 occurs only in neural tissue. Several theories were previously postulated. One was that higher brain pH than the rest of the body leads to deamidation. However, other publications [8] have shown that the brain’s pH is lower on average than the rest of the body. The average pH in the brain is 7.05, and the average pH of the body falls within the range of 7.35–7.458 [8]. Another postulated theory was that deamidation was caused by a lower turnover rate of proteins in non-dividing cells such as neurons [7]. However, the same author looked at deamidation in heart muscle cells, which are also non-dividing, just like neurons, and found that the deamidation rate in heart muscle cells is insignificant [7].

The question is, what is the real cause of 4E-BP2 deamidation in the brain? Discovering the fundamental neurobiological mechanism behind 4E-BP2 deamidation is of crucial and seminal importance for the following reasons:

1. 4E-BP2 deamidation is critical to the inner machinations of human consciousness because it significantly changes the brain’s translation (protein production) rates [6]. 4E-BP2 is also directly linked to the fundamental biological mechanism of memory [9].
2. It will be crucial to developing therapeutics against Alzheimer’s and Sclerosis because of the following:
  - The absence of deamidated 4E-BP2 in the brain leads to memory impairment [9].
  - 4E-BP2 is essential in developing long-term synaptic plasticity and social behavior [10].
  - Deamidation is related to aggregation and amyloid plaque formation, which is the root cause of memory loss in Alzheimer’s and many other neurodegenerative diseases. Deamidation accelerates amyloid formation and alters amylin fiber structure [11].
3. It is indispensable to the survival of the mammalian brain because 4E-BP2 deamidation has been conserved in all mammals for 90 million years. Therefore, this function is crucial to all mammalian brains [12].

In summary, since 4E-BP2 controls protein production in the brain, it plays a crucial role in the progression of neurodegenerative diseases. When undergoing deamidation, traditionally linked to oxidative stress, 4E-BP2 can no longer bind the eIF4E protein, fundamentally changing translation in the nervous system. This is why discovering the cause of 4E-BP2 deamidation in the brain is paramount.

Therefore, the purpose of this paper is to (1) describe a newly developed fundamental theory of the deamidation mechanism that demystifies 4E-BP2 deamidation as a process that occurs only in neurons, something that could not be carried out for 17 years, and (2) validate it experimentally in both the central nervous system (CNS) and the peripheral nervous system (PNS) by performing western blots of 4E-BP2 on the optic nerve, the sciatic nerve, the whole brain, the retinal ganglia, and the dorsal root ganglia (DRG) of 2-month-old male Wild-type mouse models.

My hypothesis, which was to be proven, is the following:

4E-BP2 deamidation happens only in neurons because of the axons and their unique properties.

## 2. Results

### 2.1. Experiments in the Whole Brain, the Optic Nerve, and the Retina of the Central Nervous System (CNS)

The first goal of the experiments was to compare 4E-BP2 deamidation in the two parts of the neurons of the brain:

(a) The axons.
(b) The cell bodies (soma).

The first tests were performed on the central nervous system (CNS) because the protein in question, 4E-BP2, is the most prevalent 4E-BP protein in the brain [6]. One specific nerve of the CNS needed to be isolated to study the prevalence of 4E-BP2 deamidation in the axon compared with its respective ganglia (soma). It would reflect where 4E-BP2 deamidation is most prevalent in myelinated neurons, the dominant type of neurons in the CNS [13]. The optic nerve was chosen to be studied in this paper because it is a large nerve of the mouse CNS, isolatable via dissection. The retina was also dissected for comparison because it is where the optic nerve axons’ soma (retinal ganglia) are located. Another reason why the tests were performed specifically on the optic nerve and the retina is because they are extensions of the brain. During embryonic development, they arise from the diencephalon, a brain division [14,15,16]. These were the first-ever experiments in which the optic nerve and the retina were dissected to detect 4E-BP2 deamidation.

**Figure 1:**
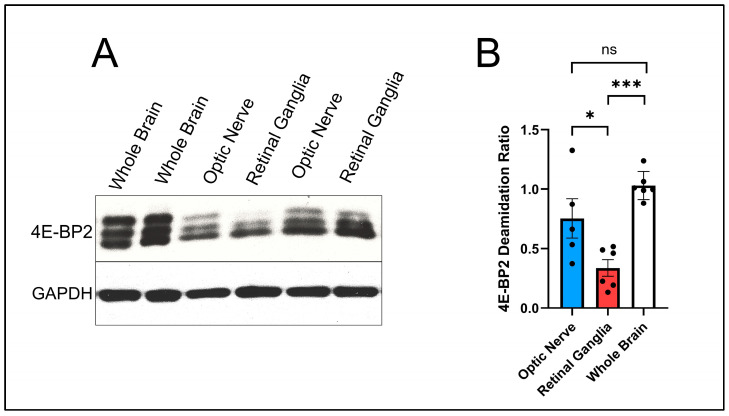
4E-BP2 western blot of the whole brain, the optic nerve, and the retinal ganglia from 2-month-old WT mice, using GAPDH as the control: (**A**) Immunoblotting data. (**B**) Bonferroni multiple comparisons test of the deamidation ratios of the three organs studied. The star (“*”) between columns symbolizes a significant difference with a *p*-value of less than 0.05 between results, whereas “ns” stands for “not significant”. Three stars (“***”) between columns symbolize a significant difference with a *p*-value of less than 0.001 between results. More stars mean a more substantial difference between results.

The second goal of the experiments was to compare 4E-BP2 deamidation in the whole brain (a mixture of axons and cell bodies) with pure axons in the optic nerve and cell body-enriched tissue from the retina. This comparison would shed light on where precisely 4E-BP2 deamidation originates. The results are shown in Figure 1.

The deamidation ratio was calculated for each region studied to accurately compare the level of deamidation between the axon-enriched brain, the optic nerve, and the cell body-enriched retinal ganglia.

For this study, a *p*-value threshold of 0.05 was used for statistical significance. Any comparative analysis with a *p*-value lower than 0.05 represents a statistically significant result (see materials and methods section).

The calculations are the following:

1. The optic nerve was found to have a deamidation ratio of 0.7539 based on five mice samples (N = 5).
2. The retinal ganglia were found to have a deamidation ratio of 0.3365 based on six mice samples (N = 6).
3. The axon-enriched whole brain was found to have a deamidation ratio of 1.0302 based on six mice samples (N = 6).

Bonferroni’s multiple comparisons test [17] proved critical when analyzing these three sample regions (see materials and methods section). As a result of these calculations, the findings from Figure 1 are the following:

1. 4E-BP2 deamidation in the optic nerve axons is significantly higher than in the soma cell body-enriched retinal ganglia (*p*-value = 0.0312).
2. 4E-BP2 deamidation in the optic nerve axons shows no significant difference compared with deamidation in the axon-enriched whole brain (*p*-value = 0.2116).
3. 4E-BP2 deamidation in the cell body-enriched retinal ganglia is significantly lower when compared with deamidation in the axon-enriched whole brain (*p*-value = 0.0004).

Based on these findings from Figure 1, the conclusion is that deamidation in the central nervous system comes from the axons.

### 2.2. Experiments in the Sciatic Nerve and the Dorsal Root Ganglia (DRG) of the Peripheral Nervous System (PNS)

The goal of these experiments was to compare 4E-BP2 deamidation in the two parts of the neurons of the periphery of the body:

(a) The axons.
(b) The cell bodies (soma).

The tests in the PNS were performed to validate that 4E-BP2 deamidation is also axon-specific in this particular system, where nerves are far away from the brain. Like the CNS, one specific nerve of the PNS needed to be isolated to accurately study the prevalence of 4E-BP2 deamidation in the axon compared with its respective ganglia (soma).

**Figure 2:**
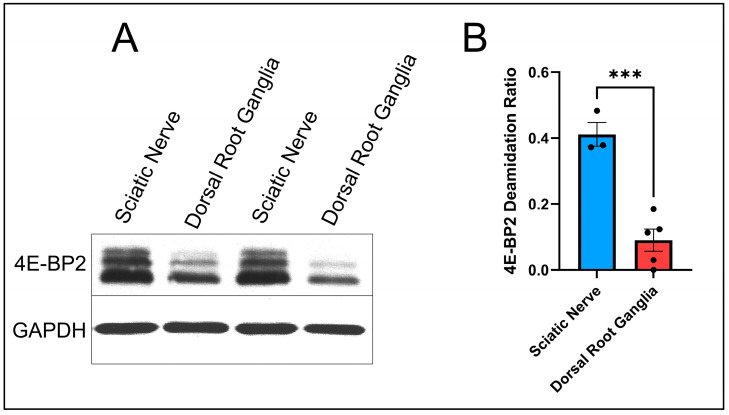
4E-BP2 western blot of the sciatic nerve and the dorsal root ganglia (DRG) from 2-month-old WT mice, using GAPDH as the control: (**A**) Immunoblotting data. (**B**) *T*-test comparing the two organs’ deamidation ratios. Three stars (“***”) between columns symbolize a significant difference between results with a *p*-value of less than 0.001.

The sciatic nerve and its soma, located in the DRG, were chosen to be studied because the sciatic nerve is the largest and longest nerve of the mouse PNS and is isolatable via dissection [18]. These were the first-ever experiments in which the sciatic nerve was dissected to detect 4E-BP2 deamidation. The results are shown in Figure 2.

The calculations of the deamidation ratios (as described previously) are the following:

1. The sciatic nerve has a deamidation ratio of 0.4112 based on three mice samples (N = 3).
2. The DRG have a deamidation ratio of 0.0902 based on five mice samples (N = 5).

Based on a statistical *t*-test, Figure 2 shows that 4E-BP2 deamidation in the sciatic nerve axons is significantly higher than in the soma cell body-enriched DRG (*p*-value = 0.0008).

Based on this finding from Figure 2, the conclusion is that 4E-BP2 deamidation in the PNS comes from the axons. The PNS results corroborate the CNS findings.

An important observation is noted when comparing Figure 1 and Figure 2: the longer the axon, the more significant the average difference in deamidation. The average length of the optic nerve in the mouse is 5 mm [19], and the mouse sciatic nerve length range is 22.6 ± 1.62 mm [20]. When looking at the difference in deamidation of 4E-BP2 between axons and soma, it was found that deamidation in the axon tissue of the optic nerve, which is fully myelinated according to previous studies [21], is 2.24 times more significant than in the retinal ganglia, based on the results in Figure 1. However, when comparing 4E-BP2 deamidation between the sciatic nerve and the DRG, it was found that sciatic nerve deamidation is 4.56 times higher in the axons.

From these results, it was concluded that a significant increase in the neuron’s length, from 5 mm to around 22.6 mm, more than doubles the difference in 4E-BP2 deamidation ratios between axons and soma.

**Figure 3:**
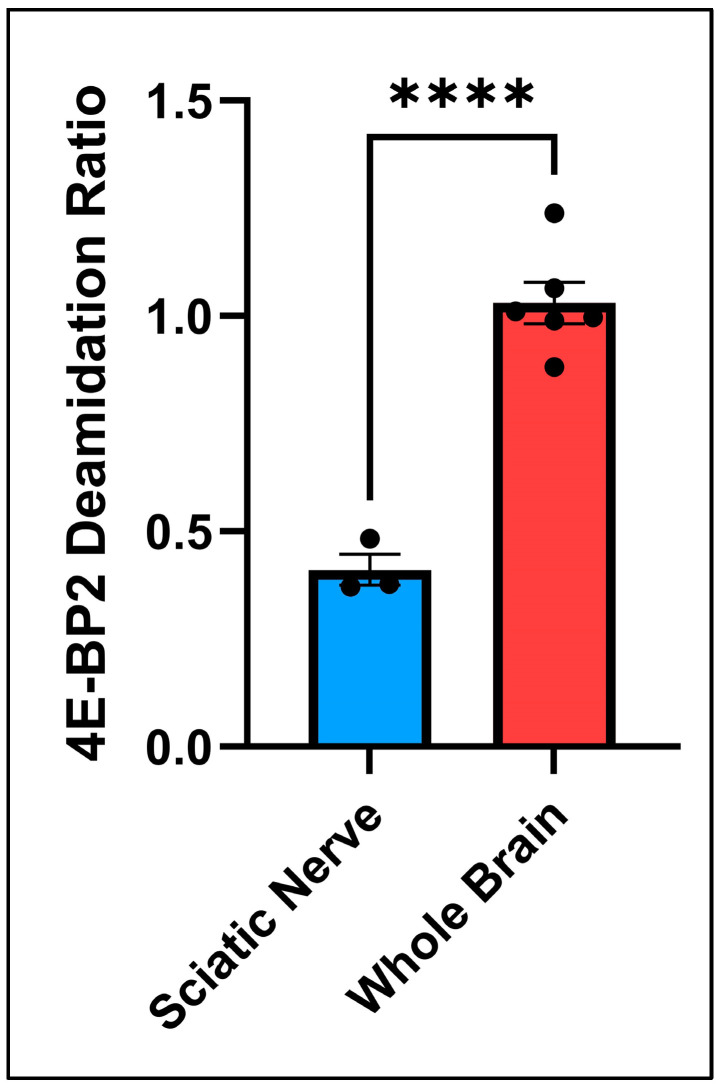
*T*-test comparison between the deamidation ratios of the whole brain and the sciatic nerve. Four stars (“****”) between columns symbolize a significant difference between results with a *p*-value of less than 0.0001.

The rates of deamidation were compared between the sciatic nerve, where axons are mostly unmyelinated [22] (not isolated), and the whole brain, where axons are mostly myelinated [23,24] (isolated), to determine the effect of myelination on deamidation because myelin degeneration is crucial in many neurodegenerative diseases, such as multiple sclerosis [25]. The results are shown in Figure 3.

Based on the comparison between the sciatic nerve and whole brain 4E-BP2 deamidation in Figure 3, the conclusion is that deamidated 4E-BP2 is significantly more enriched in the myelinated axons of the whole brain compared with the sciatic nerve’s mostly unmyelinated axons (*p*-value < 0.0001).

## 3. Discussion

Based on the above experimental results and their analysis, the following conclusions can be drawn:

1. Deamidation in the central nervous system (CNS) comes from the axons.
2. Deamidation in the peripheral nervous system (PNS) comes from the axons.
3. Deamidated 4E-BP2 is more enriched in the myelinated axons of the whole brain than in the sciatic nerve’s mostly unmyelinated axons.

The theory behind these conclusions is discussed below.

### 3.1. Half-Life Length: The Cause of 4E-BP2 Deamidation Being Neuron-Specific

My literature analysis reveals that deamidation is a spontaneous reaction, and no enzyme is necessary for deamidation [6]. Each asparagine in proteins is found to have a deamidation half-life, after which more than 50% of said asparagines are converted into aspartic acids [26]. It has also been determined [27] that deamidation depends on the exposure of the asparagine or glutamine to the solvent. The protein structure and the flanking amino acid residues determine this exposure. Based on the above literature, I concluded that the smaller the amino acid residues flanking the specified asparagine in 4E-BP2, the more this asparagine is exposed to the solvent. Also, the more the asparagine is exposed to the surrounding solvent, the shorter the half-life of the asparagine will be before it is converted into an aspartic acid. For instance, it has been found, based on Robinson’s deamidation algorithms, that Asn 26 in the ESNGP amino acid sequence of the SOD1 protein has a half-life of 71 days. In contrast, Asn 53 in the GDNTA amino acid sequence of the same protein, less exposed to the solvent, has a half-life of 17,127 days [26,27,28].

I postulated that the deamidation half-life of asparagines 99 and 102 in the NNLNNLNNH amino acid sequence of 4E-BP2 is reached in neurons after 18 days because this is consistently the amount of time required for deamidation to be significantly observed [6].

I concluded that different exposures to the solvent could not be the cause of 4E-BP2 deamidation being specific to neural tissue because 4E-BP2 proteins all have the same disordered structure [29], and the exposure to the solvent is, therefore, identical in all 4E-BP2 proteins.

Based on my analysis of the literature above, I also inferred that 4E-BP2 deamidation could not be possible unless its protein half-life was longer than the deamidation half-life of the asparagines. For instance, a 4E-BP2 protein that exists for only a few hours does not have the protein half-life necessary for detecting deamidation because deamidation can only be observed after 18 days. I therefore concluded that deamidation only occurs in 4E-BP2 found in neural tissue because the protein half-life of 4E-BP2 is longer than the deamidation half-life of asparagines 99 and 102 only in neural tissue, allowing deamidation to occur in 4E-BP2 in this specific type of tissue in the body.

### 3.2. Axons: The Cause of the Increase in 4E-BP2 Half-Life in Neurons

My literature analysis reveals that the half-life of proteins other than 4E-BP2 is significantly longer in axons. For instance, superoxide dismutase proteins (SOD1) in the axons of the motor neurons of the spinal cord undergoing axonal transport had a protein half-life of over one year [30]. In contrast, the SOD1 proteins found in kidney cells had a half-life of only 100 h [31].

Previous studies have shown that 4E-BP proteins have a half-life of only 20 h outside of neurons, such as in pancreatic beta cells [32]. As I concluded previously, this proves that 4E-BP proteins in areas outside neurons have half-lives that are far too low to reach the 18-day threshold required for deamidation in Asn 99 and Asn 102 to be significantly observed. Also, 4E-BP2 proteins in neurons were found to have a considerably higher half-life than 4E-BP2 proteins in kidney cells [33].

Since my experimental results confirmed that deamidation occurs specifically in the axons of the central and peripheral nervous systems, as seen in points 1 and 2 at the beginning of this section, I concluded that 4E-BP2 deamidation happens specifically in the axon because axons significantly increase protein half-life.

### 3.3. The Proteasome-Poor Environment in Axons—The Cause of the Increase in 4E-BP2 Half-Life

My critical analysis of the literature reveals that:

- proteasome-poor environments such as erythrocytes cause significantly longer protein half-lives [34].
- Axons are also proteasome-poor compared with the soma due to ribosome biogenesis mainly being performed in the soma [35].
- Myelinated neurons have less proteasomes compared to neurons without myelin [25].

Since my experimental results confirmed that deamidated 4E-BP2 is more enriched in the myelinated axons (fewer proteasomes) of the whole brain compared to the mostly unmyelinated axons (more proteasomes) of the sciatic nerve, as seen in point 3 at the beginning of this section, I concluded that the proteasome-poor environment in the axons is what makes 4E-BP2′s half-life longer and deamidation thus possible.

### 3.4. Establishment of the Principle Behind 4E-BP2 Deamidation Based on These Findings

The experimental conclusions found in points 1, 2 and 3 at the beginning of this section as well as the subsequent theoretical findings discussed above constitute Davis Joseph’s principle on the deamidation mechanism. It was developed over two weeks, starting on 31 August 2023, and finalized on 13 September 2023, when I was 23 years old.

This principle can be concisely summarized as follows:

Due to their proteasome-poor environment, axons increase the protein half-life, which becomes more significant than the deamidation half-life of asparagines, creating neuron-specific deamidation.

This fundamental principle is of tremendous importance because, as stated above, the neural-tissue-specific deamidation process has been conserved in all mammals for 90 million years. Given 4E-BP2 and deamidation’s role in memory, synaptic plasticity, and protein synthesis, the principle paves the way to developing multiple treatments for neurodegenerative diseases.

This principle also paves the way for monumental research on possible treatments for Parkinson’s by tying 4E-BP2 deamidation to the axon’s proteasome-poor nature because, as seen in previous literature [36], the first sign of Parkinson’s progression is the degradation of motor axons, which is tied to a change in protein production.

The results of this paper are seminal because, by proving that 4E-BP2 deamidation is found mainly in the axons, they explain why deamidation of 4E-BP2 occurs only in neurons and why 4E-BP2 deamidation is also detected in muscles. As observed in a previous publication [37], each muscle cell is associated with one neuromuscular junction (NMJ), which contains a motor axon terminal. Considering this literature information, my theory explains that deamidation was found in muscles previously not because of the muscle cell itself but because of the motor axon terminal attached to the NMJ of each muscle fiber. My conclusion that the axon connected to the NMJ causes 4E-BP2 deamidation in the muscles is supported by previous literature that shows that the concentration of NMJ axon terminals is immense in the muscles, for example, in the bicep, where 172,000 to 400,000 muscle fibers are located. Each muscle fiber has its corresponding NMJ axon terminal [38,39]. My results in Figure 2 demonstrate that the sciatic nerve and its axon terminals, which, according to previous literature [40], are connected to muscle fibers by numerous NMJs, are where 4E-BP2 deamidation is produced.

Thus, my experimental and theoretical conclusions are corroborated by 4E-BP2 deamidation previously detected in skeletal muscles [7], because skeletal muscles have a very high density of axon terminals found in the neuromuscular junctions [38]. Also, contrary to a previous literature study where the conclusion was that 4E-BP2 deamidation came from neurons and muscle cells [7], my work shows that the deamidation of 4E-BP2 found in muscles is coming from the axon terminal linked to the muscle fibers via the NMJ. The previous study used synaptophysin antibody experiments to study the presence of synapses (axon terminals) in muscle cells. The study claimed that no synapses in the muscles were found after dissection. However, the method was incorrect because the muscles were not permeabilized with Triton X-100 and treated with Cd^2+^ in a Ca^2+^-free medium before applying the synaptophysin antibody, as specified in the literature [41]. Based on this, the axon terminal in the NMJ cannot be detected without the permeabilization step and subsequent treatment with Cd^2+^.

This work also showed that, despite the vast difference in tissue composition, location, and function between the optic nerve and the sciatic nerve, the same deamidation process is observed in both organs, as shown in Figure 1 and Figure 2. As can be seen in these figures, although the optic nerve is entirely myelinated [21], whereas the sciatic nerve is a mixed neuron [42,43,44] containing both unmyelinated and myelinated axons, deamidation of Asn 99 and Asn 102 of the NNLNNLNNH amino-acid sequence in 4E-BP2 is mainly found in the 4E-BP2 proteins located in the axons for both nerves. My experimental results validate the principle of my study which states that deamidation is tissue-specific to neurons due to axons and their proteasome-poor environment.

There may be a little deamidation in the ganglia dissected, but this is most likely due to the dissection process, which always leaves a bit of the axon at the root of the soma and the rest of the ganglia. The key here is that the retinal ganglia sample is cell body enriched, whereas the axon samples are pure. A significant difference in deamidation was thus seen when comparing pure axon samples with cell body-enriched samples, and 4E-BP2 deamidation was found to occur mainly in the axons.

**Figure 4:**
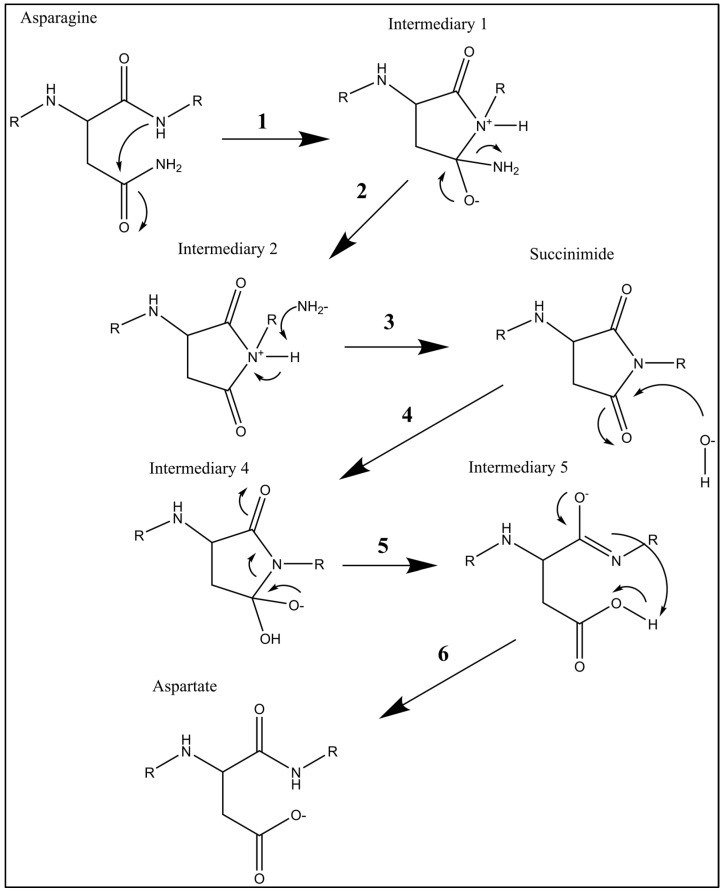
Six-step chemical reaction of deamidation occurring in 4E-BP2 at positions N99 and N102.

To explain the procedure of the chemistry behind 4E-BP2 deamidation, I invented an organic chemistry flow sheet consisting of five intermediary molecules, as seen in Figure 4. In this figure, the reaction starts spontaneously at the level of the asparagine. During this spontaneous reaction, the secondary amide’s nitrogen attacks the primary amide’s carbonyl (step 1). This creates a cycle of atoms in intermediary 1. At the level of intermediary 1, the primary amine leaves the molecule (step 2), a process known as nucleophilic substitution that creates intermediary 2. At the level of intermediary 2, the deprotonation process occurs at the tertiary nitrogen (step 3), creating a succinimide molecule. At the level of succinimide, amide hydrolysis occurs (step 4), which produces intermediary 4. At the level of intermediary 4, the bond between the nitrogen and the carbon inside the succinimide ring is severed (step 5), which produces intermediary 5. At the level of intermediary 5, an intramolecular deprotonation (step 6) produces the aspartate (the ionized version of aspartic acid) under basic conditions, which is the last step of the reaction.

Based on Figure 4, it can be said that deamidation is an oxidation reaction. This study’s results, therefore, confirm that the axon’s nature causes oxidation in translational control proteins. This means that axons may be vulnerable to oxidative stress more than any other region in cells and may also be the precursors of oxidative stress within the nervous system. My discovery of axon-induced oxidation in translational control proteins paves the way for seminal research into oxidative stress-related drug development against neurodegeneration.

## 4. Materials and Methods

### 4.1. Animal Tissue Sample Collection

My project identification code is MCGL-5205. The McGill Animal Care committee approved it on 30 August 2023. The mouse strain used is C57BL/6 (Controls WT in-house).

I invented my method of dissecting the optic nerve, the retina, and the whole brain simultaneously without relying on previous literature. The technique consists of exposing the mouse to isoflurane before euthanizing it in a carbon dioxide chamber, followed by head decapitation. I proceeded by peeling off the mouse skin and then the skull. I also lifted the whole brain from the base of the skull to expose the chiasma. Using a micro tweezer and micro scissors, I cut right between the chiasma and its connection to the diencephalon. After extracting the whole brain, I cut both connections between the optic nerve and the mouse retina.

After isolating the optic nerve, I placed the mouse eyes on Wattman paper to keep them in place and isolated the retina using a scalpel.

I also invented my unique way of dissecting the sciatic nerve without relying on previous literature by severing the mouse leg using large scissors, peeling off the skin, followed by the posterior thigh muscles to isolate the targeted nerve.

DRG extraction was performed based on my analysis of the protocol found in previous literature [45].

### 4.2. Immunoblotting

Total 4E-BP2 immunoblotting was performed on the following five organs extracted via dissection from 2-month-old Wild-type male mice: the retinal ganglia, the optic nerve, the DRG, the sciatic nerve, and the whole brain. The immunoblotting process was partly conducted as described in previous literature [33], along with several unique modifications made by the author.

The mice were given access to food and water until they reached the required age for dissection. They were in an aseptic, alternating night and day environment in the animal care facility of my lab. Mice were given isoflurane anesthesia before being euthanized in a carbon dioxide chamber, as described above. Tissue samples from the optic nerve, the sciatic nerve, the retinal ganglia, the DRG, and the whole brain were collected. Mouse tissue was lysed and homogenized using RIPA buffer (150 mM sodium chloride, 1.0% NP-40, 0.5% sodium deoxycholate, 0.1% SDS, 50 mM Tris, pH 8.0) mixed with protease and phosphatase inhibitors (Thermo Fisher Scientific, Waltham, MA, USA). Samples were centrifuged for 20 min at 16,000 RCF (Relative Centrifugal Force) at 4 °C. Immunoblotting was performed with the supernatant after the protein concentration was determined with the Bradford method. A total of 30 µg of protein was mixed with Laemmli sample buffer (50 mM Tris, pH 6.8, 100 mM DTT, 2% SDS, 10% glycerol, 0.1% bromophenol blue). The mixture was heated at 100 °C for 5 min and then resolved on a polyacrylamide gel. Proteins were transferred using a 0.2 µm nitrocellulose membrane (Bio-Rad, Hercules, CA, USA). After transfer, the membrane was blocked at room temperature using 5% milk for 1 h. The membrane was then incubated overnight at 4 °C using a total 4E-BP2 primary antibody in 5% milk. The membrane was washed with TBS-T three times before applying the goat anti-rabbit secondary antibody (Catalog No. 32460, Thermo Fisher Scientific, Waltham, MA, USA) for one hour at room temperature. Proteins were covered in ECL western blotting substrate (Catalog No. 32106, Thermo Fisher Scientific, Waltham, MA, USA) twice for 1 min and then exposed to X-ray films (Catalog No. 34091, Thermo Fisher Scientific, Waltham, MA, USA).

### 4.3. The Joseph Ratio

The intensity of the bottom band of 4E-BP2 represents the amount of non-deamidated 4E-BP2. In contrast, the intensity of the top band represents the amount of fully deamidated 4E-BP2 (deamidation having occurred in both Asn 99 and Asn 102). The Joseph ratio is the deamidation ratio I created. It is the amount of deamidated 4E-BP2 (the top band) divided by the amount of non-deamidated 4E-BP2 (the bottom band).

### 4.4. Statistical Analysis

The calculation results were obtained using the Bonferroni multiple comparisons statistical test between the three sample regions. The Bonferroni correction is performed to avoid a type 1 statistical error, misinterpreting the data and concluding that there was a statistical difference when there was no difference [17]. This article uses a probability value (*p*-value), which is the probability that the results occurred randomly [46]. A *p*-value of 0.04 would mean that there was a 4% probability that the results occurred by chance. A *p*-value of less than 0.05 represents a statistically significant result.

## 5. Conclusions

This work discovered the neurobiological mechanism of deamidation for the first time. This mechanism’s key factor is the axon, which causes 4E-BP2 deamidation due to its proteasome-poor environment. This environment increases the 4E-BP2 half-life, making it longer than Asn 99 and Asn 102 deamidation half-lives, thus assuring deamidation. This work also discovered axon-induced oxidation in translational control proteins.

To achieve these results, new dissection methods were invented for the optic nerve, the retina and the sciatic nerve.

The mechanism was then validated by conducting 4E-BP2 western blots in five different organs, including the optic nerve, the sciatic nerve, and the retinal ganglia, in which the western blots were the first-ever to be performed to detect 4E-BP2 deamidation. The *p*-values obtained from the experimental results confirmed beyond doubt that deamidation is significantly higher in axons than in cell-body-enriched ganglia in both the central and peripheral nervous systems.

These results indicate that the axons cause 4E-BP2 deamidation due to their unique proteasome-poor environment properties, thus validating my initial hypothesis on the role of axons in 4E-BP2 deamidation described in the introduction.

This experimentally validated discovery of the link between 4E-BP2 deamidation and axons is of seminal importance because it explains what causes a post-translational modification in the brain of all mammalian species, which has been conserved for over 90 million years. It paves the way to developing effective treatments against neurodegenerative diseases, such as Alzheimer’s, Parkinson’s, and many others.

This finding allowed me to discover what causes 4E-BP2 to be in much higher concentration in the brain than other 4E-BP-type proteins. This finding also allowed me to discover and prepare a new and effective treatment for memory loss. Both subsequent findings are on the verge of publication.

**Figure 5:**
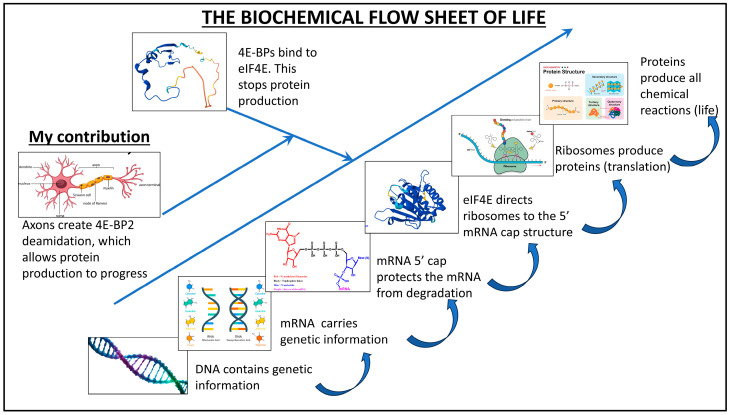
Biochemical flow sheet of the impact of the axon on protein production in the mammalian organism (all images used are royalty-free, except for the 5′ cap structure image, which the author made, and the 4E-BP and eIF4E images obtained using AlphaFold [47,48,49]).

My finding revolutionizes humanity’s understanding of protein production in the human body. Figure 5 shows a flow sheet describing protein production in mammalian organisms; DNA contains the genetic information carried by messenger RNA (mRNA). eIF4E binds to the 5′ mRNA cap structure, leading to the ribosome’s recruitment. The ribosome then produces proteins. 4E-BP proteins inhibit overall protein production by binding to eIF4E.

My contribution fundamentally changes our understanding of translational regulation by demonstrating that the axon is a protein synthesis regulator. The axon causes deamidation in the 4E-BP2 protein and, by doing so, stops 4E-BP2 from inhibiting protein production in the nervous system.

## Data Availability

The original contributions presented in this study are included in the article. Further inquiries can be directed to the corresponding author.
